# Voluntary versus State-Supported Hospitals

**Published:** 1888-04-14

**Authors:** 


### Voluntary versus State-Supported Hospitals.

By the Right Hon. the Earl of Rosebery, K.T., being the Stratford Speech specially revised.

It has always seemed to me that one of the greatest facts that the thinkers and politicians of the present day have to deal with is this great shapeless, thickly, densely pop ulated mass, which we call London. When you come to think that there are 5,000,000 of inhabitants, more or less, piled up in brick houses which, more or less, belong to them—rather less than more; more or less weather proof—rather, again, less than more, all going their different ways, pursuing their different avocations, eating, drinking, and sleeping without much relation to each other, without much individuality or community of thought, I think you will feel that there is a very considerable problem before us. To this you must add that this population is increasing at the rate of 22 per cent. That is about 1,000,000 every ten years, and some of you who read this may live to see the London which we know—a great wilderness of whited-brown brick—inhabited by 10,000,000 people. But, without looking so far forward as that, we have a very great problem to deal with now. The physical facts relating to this metropolis are sufficient in themselves. The food supply, the water supply, the drainage, all these are matters which are almost stupefying when you come to think them out. What they will be in that future to which I have attempted to call your attention the mind can hardly grasp. But even as it is now it seems to me that the problem is sufficiently worthy of your attention. We have here not a town in the sense that we meet with towns in other parts of England. It is more a series or number of communities which have, as it were, arrived at the same spot by accident. They are not a population which are entirely homogeneous, or knows much about each other, but a wayward movement of different populations that have arrived at the same area.

The question that we all ask ourselves is, What is it all drifting to? Some readers may have seen the launching of a great ship. There is a moment of anticipation when the ship seems to tremble in the balance, and one can hardly tell in what direction it is going, whether it is going straight into the river or one side, or where the great mass may direct itself. And I think those who regard the population of this great city of London must be actuated by this sort of feeling. You see it, you know that it is there, but you cannot tell whither it is going, whether it will flow smoothly down the stream of time, or whether it will become a waterlogged mass to become an engine of confusion. I cannot help thinking that when we boast, as we very often do, that this is the greatest city the world has ever seen, we forget that without administration, without organisation, without arrangement, this city may lose all the charm and all the character that it is supposed to possess, and become a mere shapeless mass of luxury and misery, of poverty and wealth, of crime and discontent and suffering. You may ask yourselves why I should begin with all these general reflections; but the fact is, they are extremely pertinent to the subject we have to plead—the cause of the hospitals of London. These institutions have a very distinct and simple office to render, and they have also a higher office, and a less simple one, which is that they form a cause and a ground on which and for which persons of all classes and ranks in the community living in the many parts of this area of London, can meet together and heartily co-operate for the public weal. If it were not, indeed, for hospitals and for places of worship, it is hard to see how this community of London would meet together, and how it could act together; but, as it is, we are fortunate in having in this city great hospitals supported by voluntary contributions, which are, as it were, the object and mainspring of a great deal of the highest effort that we know of in the course of our experience.

Of course, everybody has a good word to say for hospitals. No one can say one word against the principle of hospitals. In a catholic and unselfish spirit they relieve and save all persons distressed by illness who apply to them. I sometimes wish they were not so popular and universally well spoken of, because I believe that such is the pugnacity of the English race that, if hospitals were once well attacked, whether by politicians or opponents or anything of the sort, there would be an instant and strenuous movement to assist them. But because everybody says a hospital is an excellent thing, there is a sort of apathy about a generally accepted truism of every sort, people remain quiescent, and allow others to come to the rescue. To encourage that feeling of pugnacity I may at least say that the hospitals are fighting at this moment a strenuous battle against that great giant or devil, whichever you care to call it, of poverty, and we all know what a difficult fight that is. If some of those people who hold aloof from the movement were to walk through some of the wards of our hospitals which have been emptied for want of funds, and could see the beds rolled up and the furniture covered up, and all the appliances of relief piled away useless, like spiked guns, they would be inclined to open their pockets for a moment and to awaken the dormant machinery of mercy before them. But supposing we do not succeed in awakening the sympathies of the people by such a sight as I have illustrated, or by the sermons of ministers of religion, can we continue with an annual deficit every year in regard to the hospitals? You know very well what the natural result must be. These hospitals must come upon the rates. I am not in favour of hospitals coming upon the rates. I am in favour of voluntary-supported hospitals as against rate-supported hospitals. I will tell you one primary and simple reason. I daresay you will not agree with me, but I will risk it—it is that the rates are high enough already.

Taking another ground, I should say that we have always been proud in this country, in comparing our position with that of other countries, that our hospitals are supported, not by rates or taxes in the main, but by the voluntary effort of our own population. I think that is a thing to be proud of. I will give another reason. I would not lose this great source of voluntary effort and voluntary communion between the different classes of the community. I believe that the best of all energy, the best of all motive power, is voluntary effort. You cannot buy zeal or enthusiasm, and yet zeal and enthusiasm are only the extreme form of voluntary effort. Let us remember what can be done by voluntary effort. When the great Dr. Chalmers, a preacher who is a prophet in Scotland, and who will never be forgotten so long as Christianity is remembered in the land, took charge of a most populous parish in Glagow, he said to the Town Council, "Your poor relief is costing you in this parish £1,400 a-year. You hand the poor over to me and I will not charge you anything." The Town Council were extremely glad to acquiesce, and at once closed with this offer. He mapped out the whole district into small areas, controlled by voluntary officers, who found out the impostors in the district and the proud poor who had never asked relief from the parish. He asked his church congregation to assist him by their contributions, and in four years he had reduced the cost from £1,400 to £280 a-year, while he relieved all the deserving poor and simply let the imposters go free. But there is another reason why I think we should specially appreciate voluntary effort in connexion with this particular movement. Where subscriptions are voluntary, subscribers will look out very closely to see that their money is well spent. A man will not go on subscribing annually to any institution, however great its name and reputation, without seeing how his money is spent. There is always a little wrench about parting with money, and when the parting comes periodically a man is much more sensible to the wrench if he have a doubt about the object to which the money is devoted. You find, therefore, that voluntary hospitals are closely looked after by persons who make it their duty to see that the money is well spent.

But is that the case with rate-supported hospitals? No. I bring no general allegation, but I say it is at least probable that the expenditure will not be so closely looked after as in voluntary hospitals, because the rate-payers' business is everybody's business, and everybody's business is nobody's business. You know what took place at the Eastern Hospital, one of London's state-supported infectious hospitals. It was a place flowing with milk and honey, or rather with Champagne and Claret and Burgundy, and things which somebody liked better than milk and honey. It was consequently found that the patients there cost three times as much as those in any other hospital. The result of a short inquiry held as regards the officials paid out of the rates was that the superintendent resigned, the clerk of the committee absconded, the medical superintendent was suspended, and the clerk of the managers was cautioned. That puts in a sufficiently glaring way to appeal to any common sense the difference between a hospital supported by rates, the expenditure of which no one particularly cares to look after, and that supported by voluntary subscriptions, which it is the interest of every subscriber to see well spent. I do not pretend to say that all the rate-paid hospitals are like this; but what I do impress on you is that there is more risk of jobbery, and extravagance, and culpable management in a rate-supported than in a voluntary hospital.

There is another point in this connexion. I think there is more individuality in the voluntary system than in the other. Individuality is a very long word, and I do not know that it is perfectly understood in London, because in London my firm impression is that we have less of it than in any other city in the world. But what I mean is this, that in the voluntary hospital every single case is more or less likely to be treated on its own merits, and with a greater interest than in a great official, rate-supported hospital. Let me give you an illustration: There was a great foundling hospital in Russia; it was a tremendous affair, controlled by the Government, and so perfectly organised that every wet nurse had her own uniform, and that uniform corresponded with that of the regiment into which the foundling was meant to go if he ever lived to go into a regiment at all. And it is said that at a given word of command every foundling baby simultaneously imbibed nourishment at nature's fount. The discipline was perfect, but the babies died.

I am therefore in favour of voluntary hospitals as against state-paid hospitals. I have not by any means exhausted the reasons I have, but you are not expected to exhaust a case in a speech like this. But if you wish this system to be pursued there is one absolute necessity, and that is subscriptions. I am afraid that, if we do not get sufficient subscriptions, all our reasons in favour of the excellence of voluntary hospitals will fall to the ground, and the hospitals will be thrown back upon the rates. This is a question of very great importance to all classes of the population of London; it is important to all classes because no class is exempt from suffering, and while the rich benefit by the skill acquired by doctors in hospitals, the poor use those hospitals themselves. That, I think, is not an unfair division of the benefits accruing from hospitals. But in order to continue this system, we want not merely subscriptions but public opinion. The working classes of this metropolis have not, I dare say, a great deal of spare cash to give to hospitals. I never knew of anybody who had spare cash. I believe the working classes do contribute very considerably to the maintenance and the support of hospitals. But if the working classes have no spare cash, they still have what is of infinite importance. They can make and they can wield the gigantic force of public opinion in this country. If the working classes—the masses of the population of this country and metropolis—only let it be known that they are in favour, emphatically and entirely, of these voluntary hospitals being supported, there will be no difficulty in finding the funds required. What we want is the volume of public opinion to carry us on.

